# Improving ocean reanalyses of observationally sparse regions with transfer learning

**DOI:** 10.1038/s41598-025-86374-4

**Published:** 2025-01-21

**Authors:** Simon Lentz, Sebastian Brune, Christopher Kadow, Johanna Baehr

**Affiliations:** 1 Institute of Oceanography, Center for Earth System Sustainability, Universität Hamburg, Hamburg, Germany; 2 German Climate Computing Centre, DKRZ, Hamburg, Germany

**Keywords:** Transfer learning, Convolutional U-nets, Physics-informed, Climate hindcast initialization, Ocean heat content, Physical oceanography, Climate and Earth system modelling, Computational science

## Abstract

Oceanic subsurface observations are sparse and lead to large uncertainties in any model-based estimate. We investigate the applicability of transfer learning based neural networks to reconstruct North Atlantic temperatures in times with sparse observations. Our network is trained on a time period with abundant observations to learn realistic physical behavior. Evaluating it within a consistent data assimilation framework, this network learns and reproduces its training data’s physical patterns. Additionally, the network is able to transfer these patterns towards a historical ocean heat content estimate in times with sparse observations. Consequently, with infrequent input data, machine learning reconstructions exhibit similar physical structures, while correcting for known errors compared to state-of-the-art data assimilation products. In this manner, transfer learning can impact the initialization and evaluation of climate hindcasts. Furthermore, by exhibiting the capability to accurately transfer results from high to low-frequencies, transfer learning based neural networks showcase their relevance in mixed-frequency measurements beyond climate science.

## Introduction: climate data reconstructions in observationally sparse regions

In regions with sparse observations, all earth system model (ESM) based reconstruction methods possess considerable uncertainty and errors in their representation of earth system variables^1^. Most commonly, data assimilation is used as state-of-the-art reconstruction method for reanalyses and climate hindcast initialization. In the absence of sufficient observations, ESMs are free to integrate their own biases or misrepresentations into the assimilation process^2^. Therefore, this uncertainty is inherent to data assimilation in general. In order to bridge the qualitative gap between time periods with abundant and sparse observations, an alternative approach to reconstructing sparse observations is required.

**Fig. 1:**
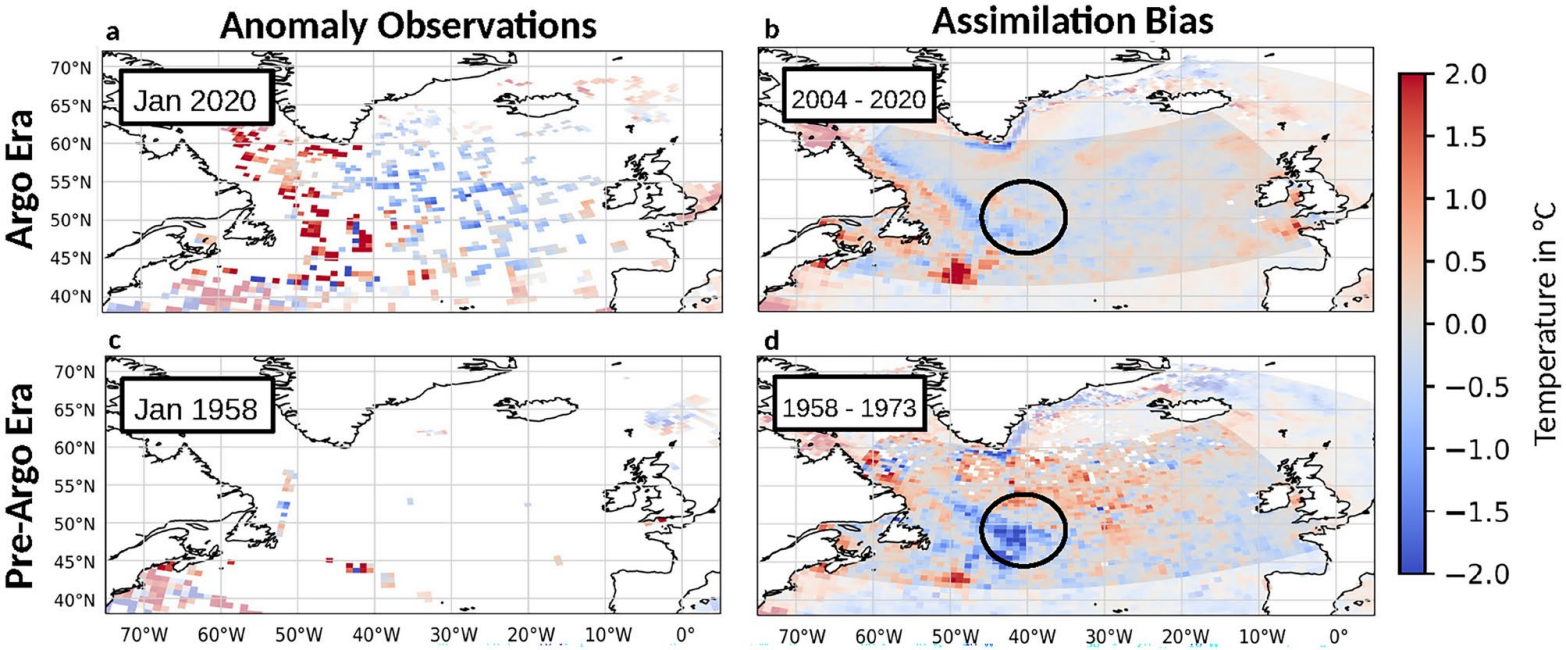
Sea surface temperature (SST) biases of the assimilation reanalysis and EN4 observations in different time periods illustrate the difficulties of data assimilation in sparsely observed regions and times. Example SST anomaly observations for the Argo and pre-Argo era from January 1958 and January 2020. The biases are taken by subtracting observations from the assimilation reanalysis SST cumulatively over a 15 year period (pre-Argo: 1958–1973, Argo: 2004–2020). During the well-observed Argo era, the assimilation reanalysis shows lower and more spatially consistent biases, while low observational density leads to much higher regional biases in unobserved regions, in particular in the area of the western North Atlantic Current (NAC). In particular the region of the NAC’s northwest corner has been highlighted by the black circle. The white pixels describe areas with no observations over the entire time period. The analysis region of the Subpolar Gyre is marked in darker colors, while the remaining NA regions are shown in faded colors. This figure was created with Python 3.10 (Matplotlib 3.5.2 [https://pypi.org/project/Cartopy/], Cartopy 0.20.0 [https://matplotlib.org/]).

The amount and quality of observations for many earth system variables can vary greatly, regionally and temporally^3^. This is especially true for inaccessible or remote areas, such as the subsurface oceans, preceding advanced and widespread measuring techniques like the Argo project^4^. As a result, the initialization of corresponding climate hindcasts is profoundly impacted by underlying ESM biases and uncertainties, as there are not sufficient observations to correct them^2^. A prominent and recurring example of such an ESM error’s influence on subsequent reanalyses is the North Atlantic Current’s (NAC) northwest corner. In particular, coupled ESMs misrepresent this turn in the western NAC, leading to SST biases of up to 10 °C^5^. High horizontal resolution models capable of resolving local eddies have been able to partially alleviate some of the biases, but only under substantially greater computational expenses^5,6^. As a state-of-the-art climate model, the Max Planck Institute’s MPI-ESM, still exhibits significant biases in the representation of the NAC’s northwest corner^7,8^. This results in large regional biases for subsequent reanalyses, such as the weakly coupled ensemble Kalman filter data assimilation product^9,10^ used in this study. The data assimilation’s 3d temperature reanalysis, which is utilized in this work as training data, evaluation data and general point of reference will from now on be referred to as assimilation reanalysis (for a more detailed description of the assimilation process and products you can refer to appendix A). The resulting bias is shown in Fig. 1. Here, the assimilation reanalysis’ accuracy is severely influenced by the quantity of its observational input. In times with high amounts of observations, the assimilation reanalysis is able to correct for its underlying ESM bias, while in earlier time periods it duplicates the ESM’s cold bias in the region of the NAC’s northwest corner.

Machine Learning has greatly impacted the climate sciences in a variety of ways, including climate pattern recognition^11^, climate modelling^12^, climate prediction^13,14^ and even climate change mitigation^15^. The ability to approximate complex systems and extract dynamic patterns enables machine learning approaches to succeed in data assimilation tasks even without deterministic knowledge of physical processes^16^. Convolutional neural networks have recently shown great promise in the reconstruction of undersampled Earth System variables^17^, especially those NNs originally developed for image inpainting^18^. By learning and applying the physical patterns of their training data, convolutional neural networks can reconstruct climate variables effectively and accurately^17^.

In addition to learning and reproducing their training data, neural networks can also be trained to transfer features or patterns to new temporal or spatial domains. This process is often referred to in the computer sciences under the broader field of transfer learning (TL)^19,20^. In the environmental or physical sciences, learning and transferring physical features of a system has recently generated a high amount of interest in the field of physics-informed neural networks (PINNs)^21–23^. PINNs learn to respect physical boundaries of a system by being taught the underlying equations either directly or implicitly. Including hard boundary conditions and equations into PINN training however requires a lot of computational resources and expertise. In a softer approach, the training data of PINNs is selected to encourage convergence towards a physically realistic solution in what is referred to as an observational bias approach to PINNs^22^.

In light of the challenges associated with data assimilation in sparsely observed regions and the potential of machine learning techniques, an alternative approach is needed. While PINNs have shown significant success in assimilating state variables, such as hydrological flow parameters^24,25^, their utilization for climate variable assimilation remains entirely unexplored. We suggest the application of transfer learning in a physics-informed manner for large-scale data assimilation of climate variables in observationally sparse regions. By combining machine learning techniques, observational data, and ESM outputs, we aim to leverage the physical patterns learned by neural networks to enhance the accuracy and efficiency of data assimilation processes. Therefore, we address the qualitative gap between data assimilation products with abundant and sparse observational input and enhance our understanding of the corresponding climate dynamics. This, in turn, also has the potential to facilitate more accurate hindcast initializations and assessments.

## Methods: transfer learning–transferring subpolar gyre ocean heat content patterns into the past

In order to provide a proof-of-concept for the potential of physics-informed approaches to enhance data assimilations in observationally sparse regions, we propose utilizing a transfer learning NN to reconstruct North Atlantic Subpolar Gyre (NA SPG) ocean heat content (OHC). Due to the substantial temporal variation of subsurface observations, OHC serves as an ideal variable to study in the NA region. Although OHC is not a directly measured variable, we decide to display OHC instead of the measured subsurface temperatures. Contrary to subsurface temperatures, OHC gives the opportunity to summarize the different subsurface temperature levels, visualize large scale patterns in 2D and is discussed much more extensively in literature (the formula to turn subsurface temperatures into OHC as well as other preprocessing steps can be found in appendix A).

Building on the approach of Kadow et al.^17^, we extend their machine learning framework to reconstruct three-dimensional subsurface temperatures in the NA SPG region. These partial convolutional U-nets have successfully reproduced dynamic temperature patterns by learning the physical connections between neighboring grid cells even with very limited input data^17^. Hence, they possess all essential capabilities for reconstructing SPG OHC during periods with sparse observations. We propose to enhance these convolutional neural networks with an observational bias physics-informed approach^22^. In line with this definition, we teach the utilized neural network (NN) realistic physical patterns by carefully selecting appropriate input data. In our case, this means training a partial convolutional U-net on the realistic physical patterns of the assimilation reanalysis during times with high observational density. Afterwards we expect the network to transfer the learned patterns towards an improved historical OHC estimate in times where observational input is sparse and the assimilation reanalysis hence flawed. However, we stay in the numerical model realm and do not constrain the machine learning algorithm by any physical laws. This avoids model instabilities when the produced data set is used as an initalization for climate predictions. To avoid confusion, we therefore refrain from calling our U-net a PINN. Instead, we refer to transfer learning using scientifically selected training data of numerically solved and therefore observed physics. Our approach can be supplemented by additional hardcoded physics-informed approaches in the future. For a more comprehensive description of the transfer-learning based network architecture (Fig. S1), training procedure and input data, please refer to the supplementary material in appendix A.

In order to increase our training data, we train the network on the monthly outputs of 14 of the assimilation reanalysis’ 16 members (while the remaining two are held back for later testing) during the Argo era from 2004-2020. The training input then consists of the assimilation reanalysis and a binary mask marking the places of observations. Additionally, a corresponding masked assimilation reanalysis, meaning the assimilation reanalysis multiplied with the binary observations mask, is produced as an input of the NN training. In this way, we gain a much more variable training input compared to utilizing just direct observations as training input. For the final reconstruction of the trained neural network, the masked assimilation reanalysis in the NN input is then replaced by the real EN4 observations.

In order for the NN to learn accurate physical patterns, choosing its training data carefully for the later transfer learning is essential. Due to sparse sub-surface oceanic observations, all reanalyses possess a relatively high uncertainty in their estimate of 20^th^ century SPG OHC. However, starting in the 2000s, this uncertainty decreases rapidly correlated with the introduction of the Argo project’s continuous measurement of subsurface temperatures in 2004^4^. Consequently, the assimilation reanalysis can be divided into two distinct time periods, when considering the observational density of NA subsurface temperatures. Pre-Argo era assimilation reanalyses (1958–2004) possess higher uncertainty and significant modelling errors, while Argo era assimilation reanalyses (2004–2020) are characterized by lower uncertainties due to the significant increase in observations.

**Fig. 2:**
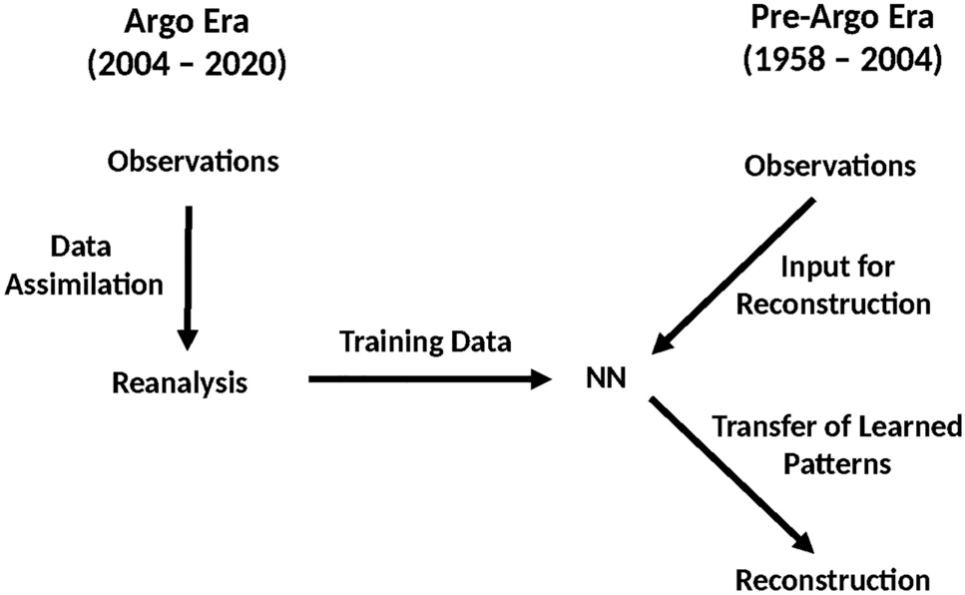
Experimental Setup: Training a our partial convolutional U-net with accurate Argo era (2004–2020) assimilation reanalyses in order to better reconstruct pre-Argo era (1958–2004) observations. During the Argo era, high amounts of observations support the assimilation process to create a complete state with low modelling errors and uncertainty. A NN trained on assimilation reanalyses from the Argo era learns realistic representations of the physical reality. Transferring these spatial patterns, it reconstructs states with low observational density during the pre-Argo era with low modelling errors.

**Fig. 3:**
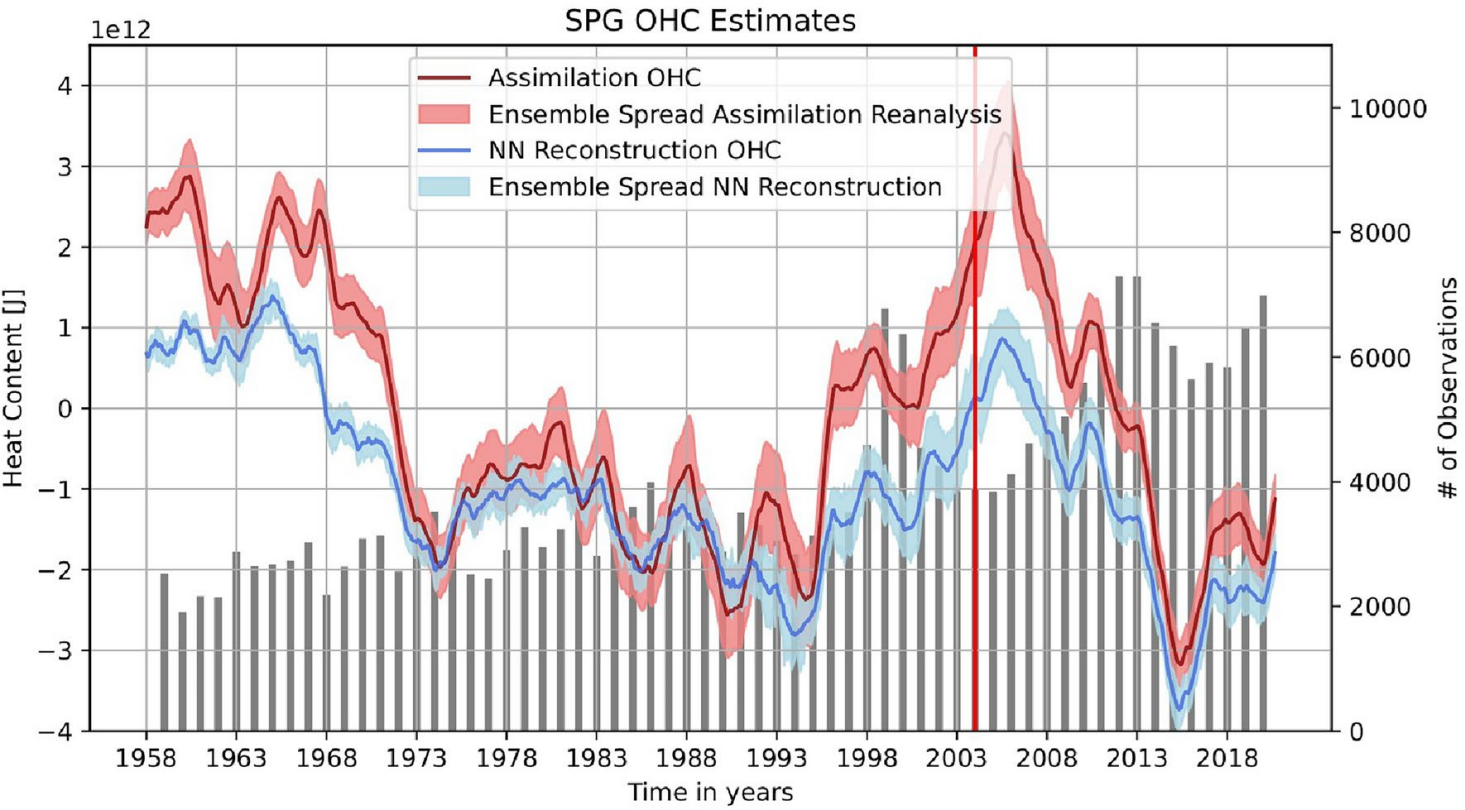
Comparison of data assimilation and NN SPG OHC timeseries. Annual running means of assimilation reanalysis (red) and NN reconstructions (blue) are shown with their corresponding ensemble spread. The NN ensemble spread is created by reconstructing all 16 assimilation reanalysis members in order to translate the training data’s uncertainty to the NN reconstructions. Additionally, the lightgrey bars show the amount of cumulative annual observations in the NA SPG. The vertical red line depicts the start of the training period (Argo era).

Our approach combines machine learning and earth system modeling (Fig. 2). Training a neural network on more accurate and less uncertain assimilation reanalyses from the Argo era, we enable it to learn highly realistic spatial patterns of NA SPG OHC. For both training and reconstruction, we use anomaly temperature fields, as we aim for our NN to reconstruct monthly variations instead of just learning the training data’s underlying climatology. As we assume the assimiliation reanalysis’ OHC estimate of the Argo era to be more accurate, only the monthly climatology from this time period was utilized to calculate the anomaly fields. We then investigate, if the network is able to transfer the learned, more accurate patterns from the Argo era when reconstructing pre-Argo era observations. This should result in reduced modeling errors and more realistic OHC estimates. The temporal division between reconstruction and training periods enables the neural network utilize the strengths of the assimilation reanalysis and apply them to counteract its weaknesses in unobserved time periods (Fig. 3).

## Results: improving assimilation reanalyses with transfer learning

The transfer learning based NN is trained on Argo era assimilation reanalyses and consequently asked to reconstruct observational 3D temperature fields with a focus on the pre-Argo era (training setup in Fig. 2). The output of the NN’s 3D temperature reconstruction will be referred to as NN reconstruction. To prevent overtraining, two assimilation members are additionally held back for independent testing. After reconstruction, the 3D temperature field is transformed into a 2D OHC field (details in appendix A). Both during and before its training period the NN is able to reproduce the assimilation reanalysis’ OHC estimate well (Fig. 3, anomaly correlation coefficient (ACC) $= 0.93$). However, during the Argo era, the agreement is much higher (ACC $> 0.99$) compared to the pre-Argo era (ACC $= 0.91$). These differences, in particular during the pre-Argo era, however, are to be expected. The systematic bias during the Argo era can be well explained by the bias of the assimilation reanalysis. If compared to the existing observations, a consistent bias of the assimilation reanalysis over all subsurface temperatures becomes apparent (appendix B, Fig. S2). By orienting itself more strongly at the observations this bias is thus not transferred to the NN reconstructions. Additionally, other reanalysis products, such as the EN4 objective analysis^26^, also point towards a weaker OHC peak around the year 2005. Thus, the NN reconstruction’s lower estimation of this OHC peak cannot be asserted as an inaccuracy. A closer examination of the assimilation reanalysis bias during the Argo era can be found in appendix B. The differences between assimilation reanalysis and NN reconstruction during the pre-Argo era, especially during the first 15 years, are less systematic, although here also a cold bias is apparent. As we know that the patterns and accuracy of the assimilation reanalysis change with decreasing observations, so does the correlation between the NN reconstructions and the assimilation reanalysis. Especially in the context of other OHC estimates, the NN reconstruction still remains close to the assimilation reanalysis (appendix B, Fig. S3). The overall agreement between both timeseries demonstrates that the NN is able to learn and reproduce the assimilation reanalysis’ OHC estimate. Visual inspection of individual reconstruction images show that the NN is also able to reproduce the complex regional patterns of the assimilation reanalysis. Examples of this reproduction can be found in appendix C (Figs. S4, S5, S6, S7, S8, S9). Additionally, we can compare both the NN reconstructions and assimilation reanalysis with an independent baseline. As there are no actual observations available during the pre-Argo era, we can only compare both estimates with a third independent OHC reanalysis. To do that, we have chosen the EN4 objective analysis^26^, in order to establish if the NN shows similarly realistic physical patterns also outside of its training period. Appendix E (Figs. S11, S12) shows that both assimilation reanalysis and NN reconstructions exhibit similar RMSE and correlation with the EN4 objective analysis during both Argo and pre-Argo era. Therefore, we can conclude that the NN is able to learn physical patterns and apply them to new anomaly observations outside of its training period. Thus, it is able to reproduce the assimilation’s state-of-the-art OHC estimate without exhibiting significant biases when reconstructing pre-Argo era observations.

**Fig. 4:**
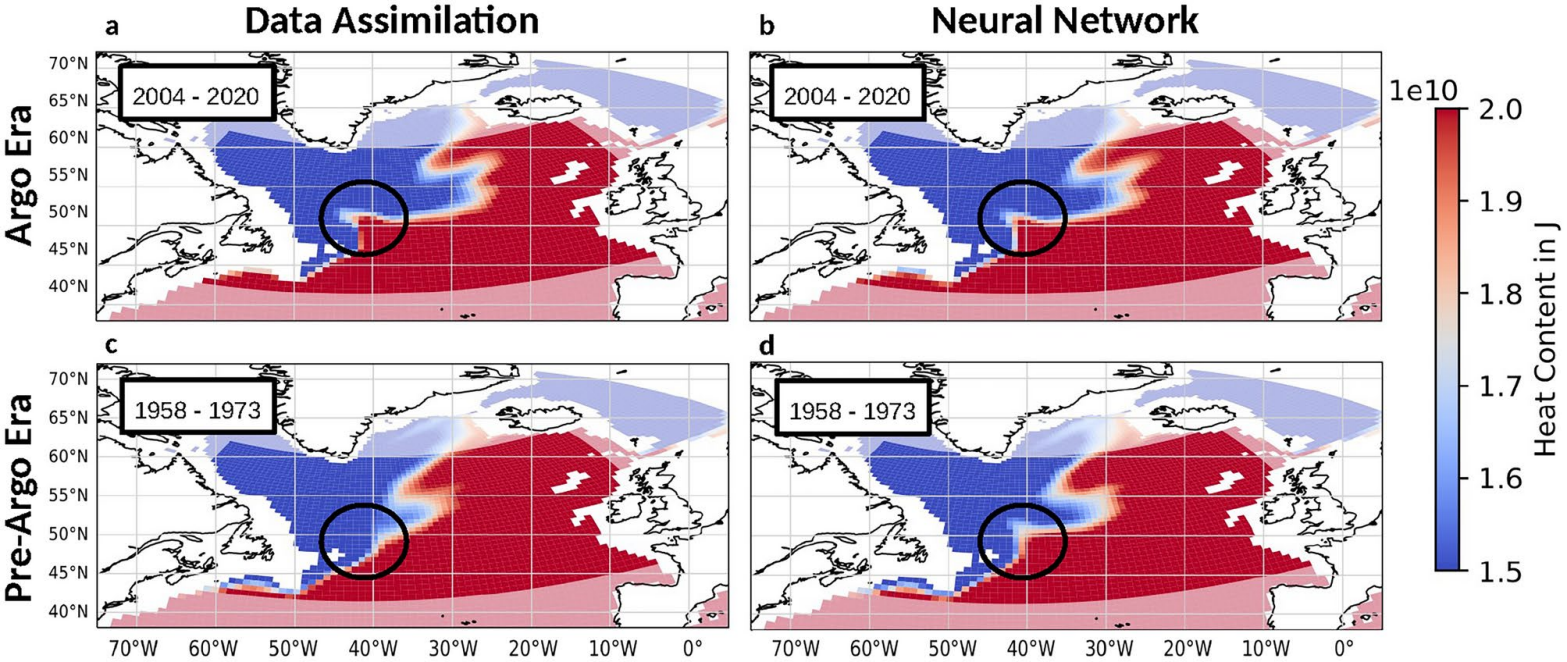
Assimilation reanalysis and NN reconstruction of the North Atlantic Current’s northwest corner. All OHC estimates are calibrated to show the NAC flow as the gradient between the red and blue areas, while the black circles show the region of the NAC northwest corner. Data assimilation (**a**) and NN reconstructions (**b**) during the later Argo era (2004–2020) show a sharp corner in the northwest of the NAC. In a comparable mean during the pre-Argo era (1958–1973), the data assimilation (**c**) misses this corner, while the NN still shows the characteristic sharp right turn (**d**). As the binary colobar of this plot is tuned to show the northwest corner, a more detailed version can be found in appendix F (Fig. S13). This figure was created with Python 3.10 (Matplotlib 3.5.2 [https://pypi.org/project/Cartopy/], Cartopy 0.20.0 [https://matplotlib.org/]).

Up to this point, we have shown that the NN manages to learn and apply complex regional patterns and thus is able to reproduce the assimilation’s physically realistic estimate during and outside of its training data. Additionally, we can now examine the differences between the OHC estimates in the pre-Argo era more closely and establish if our experimental setup can actually correct for the assimilation reanalysis’ model biases.

In this study, we assume that the assimilation reanalysis, due to its state-of-the-art EnKF method, exhibits generally more realistic patterns compared to the interpolated EN4 objective analysis. Only in specific circumstances, when the underlying model possesses a clear bias or error, does the assimilation reanalysis misrepresent the physical reality when there are no observations available to correct it. Thus, we can focus further evaluation on a known misrepresentation in the assimilation reanalysis during the pre-Argo era, the pathway of the NAC (Fig. 1). One of the NAC’s characteristic features in the NA SPG is its northwest corner. The underlying MPI-ESM of the assimilation reanalysis is not able to represent that corner correctly. Thus, without sufficient observations, the assimilation reanalysis reverts back to the model solution of a straight northeastern NAC flow^7^. During the Argo era, both the NN and assimilation reanalysis represent the northwest corner very well, guided by the amount of observations (Fig. 4a, b). In the case of the assimilation reanalysis however, the amount of observations in the SPG during the pre-Argo era, and in the first 15 years from 1958–1973 in particular, is not sufficient to counteract the underlying modelling bias. And in this case, the advantage of our approach becomes clearly visible. While the assimilation reanalysis cannot draw enough information from sparse observations leading to a misrepresented NAC flow (Fig. 4c), the NN does not make the same mistake. Thus, the NN reconstructions show the northwest corner correctly during the pre-Argo era even without the necessary observations as indicators (Fig. 4d). This assumption is also supported by the NN reconstruction’s lower RMSE with the EN4 objective analysis in the western NAC region when compared to the assimilation reanalysis’ RMSE (Fig. S11).

The NAC’s northwest corner flow has been asserted by a wide range of research also before the availability of Argo observations^27,28^. Additionally, the assimilation reanalyis’ SST bias during the pre-Argo era shown in Fig. 1 also supports this existence of the northwest corner during the pre-Argo era and its misrepresentation in the assimilation reanalysis. As the NN reconstructions directly adapt to their observational input, it is not possible to make a valid bias estimate with those same observations. However, Fig. 4 clearly proves that the NN reconstructions represent the northwest corner much more realistically in the pre-Argo era. Additionally, further investigation confirms that this difference between assimilation reanalysis and NN reconstructions only appears in the bias regions, when observations are sparse. In appendix D, the SST timeseries in the NAC’s northwest corner region show that the characteristic bias of the assimilation reanalysis appears explicitly in the months with an insufficient number of available observations (Fig. S10). This misrepresentation can also be examined in the assimilation reanalysis’ anomaly OHC estimate for individual months (Fig. S7 in appendix C.). Therefore, the NN reconstructions do more than just use a more realistic basic climatology. Instead, we can also assume that in addition to providing a more reasonable average representation, the NN reconstructions also represent the monthly variability of the northwest corner region more realistically.

Overall, we can therefore demonstrate that by learning and applying the physical patterns of its training data, a transfer learning based NN can be used to correct for assimilation products with misrepresentations and biases in time periods and regions with sparse observations. Thus, we can assume that NN reconstructions provide a more realistic estimate of the physical OHC patterns in data sparse regions and time periods.

We are convinced that further research into the applicability of physics-informed machine learning for the reconstruction of sparse observations holds great potential. In our approach, we employed a comparatively simple convolutional U-net for data assimilation tasks. More sophisticated machine learning techniques, such as the supplementation by additional spatial or temporal information, can further improve reconstruction results. Additionally, we took a soft approach to PINNs to maintain flexibility and a wide range of possible applications. Exploring the impact of supplementing the observational bias with a hardcoded physics-informed learning bias could be beneficial for more specified applications^22^. Furthermore, this work has only examined the reconstructions in their capacities as reanalyses. The effect this improved OHC estimate has on hindcast initialization has not been evaluated yet. The promising results of this study however warrant further investigation into the impact of machine learning based transfer learning on hindcast initialization, particularly in data-sparse regions. It is important to note that our method assumes stationary boundary conditions between the training and evaluation periods, which may pose challenges in a changing climate. We recognize that the North Atlantic, as every part of the climate system, is subject to internal variability, such as the amplitude of subsurface temperatures or AMOC strength^29^. However, even scarce input can provide information to the network concerning the amplitude of subsurface temperature variability. In our case, the NN especially learns the ocean dynamics and meso to large scale physical patterns, which can be assumed to be stationary over the investigated time period of 60 years, especially when they are largely influenced by topography such as the NAC’s northwest corner. Additionally, detrending anomalies before training and reconstruction can partially address the remaining changes. However, with accelerating anthropogenic climate change, it is essential to analyse its effect on reconstruction biases and consider the supplementation of the network input with physical boundary parameters. Further research could consider changing boundary conditions while also investigating the NN’s ability to improve on temporally varying model biases. Finally, the potential impact of successful transfer learning, as demonstrated by the NN in this study, on the future planning of observational measurements should be explored. As demands for higher resolutions continue to grow across various domains, including Argo floats and remote sensing, they are often constrained by available resources. If we can reliably transfer accurate results from high-frequency to low-frequency measurements using physics-informed machine learning, it could substantially influence the strategic deployment of in situ observations.

## Conclusions: a new approach to reconstructing sparse observations

We have shown that transfer learning based infilling can provide a significantly improved OHC estimate relative to traditional assimilation reanalyses when observational data is scarce. Moreover, it is computationally much more efficient and flexible in its setup compared to traditional data assimilation methods. Especially with growing datasets, increasing resolutions and larger ensembles, the necessity for resource-efficient alternatives will certainly grow. Of course due to the nature of its setup, the neural network still requires the data assimilation as training input. But as this study has shown, NN reconstruction such as ours can achieve state-of-the-art results also outside of their training period. As they additionally are much more flexible with individual tasks, such as the creation of new ensemble members or revised estimates with corrected observational input, NN reanalyses can still provide a significant reduction in computational requirements for data assimilation tasks. Therefore, transfer learning can prove valuable to the reconstruction of various variables across all regions with significant variations in observational availability over time. Given the rapid evolution of observational methodologies, be it with satellite observed SSTs or Argo observed subsurface temperatures, transfer learning can bridge the qualitative gap in data assimilation products for past time periods. Consequently, their application can have a significant impact on our ability to initialize and evaluate climate hindcasts as well as our overall understanding of sparsely observed past climatic states. Additionally, frequency variations in input data are encountered across diverse fields. This study illustrates that by reconstructing measurements with spatial and temporal variations at a consistently high level of quality, transfer learning based NNs hold the promise to influence mixed-frequency measurement challenges in a broad range of disciplines beyond the climate sciences. Consequently, we anticipate that this work stands only at the beginning of the integration of physics-informed machine learning into data assimilation, the climate sciences in general but also across the broader scientific community.

## Supplementary Information

Supplementary Information.

## Data Availability

All data generated by our NN that was utilized for this publication is openly accessible under: https://drive.google.com/drive/folders/1xjqe9aDaNa9bNDg5CJXl-AMp4qvLZmgl . All code utilized to generate this data can be found under: https://github.com/slenta/Physics-Informed-CNN-Reconstructions.
