# Supplementary material for: Improving ocean reanalyses of observationally sparse regions with transfer learning
Source: Sci Rep. 2025 Jan 21;15:2640. doi: 10.1038/s41598-025-86374-4 (PMC11751375; doi:10.1038/s41598-025-86374-4)
Supplement: Supplementary file 1 — Supplementary Information. [file 41598_2025_86374_MOESM1_ESM.pdf]

Improving Ocean Reanalyses of Observationally Sparse Regions with Transfer Learning

Simon Lentz[1*], Sebastian Brune[1†], Christopher Kadow[2†], Johanna Baehr[1†]

[1] Institute of Oceanography, Center for Earth System Sustainability, Universität Hamburg, Hamburg, Germany.
[2] German Climate Computing Centre, DKRZ, Hamburg, Germany.

* Corresponding author.
Email: simon.lentz@uni-hamburg.de
Contributing authors: sebastian.brune@uni-hamburg.de, kadow@dkrz.de, johanna.baehr@uni-hamburg.de
† These authors contributed equally to this work.

Appendix A Supplementary information

Data. In this study, we use the 3D temperature reanalysis from a 16 member weakly coupled MPI-ESM ensemble assimilation[1,2], spanning the time period 1958-2020 to train the NN to reconstruct 3D temperature profiles. In particular, we use the low resolution version MPI-ESM-LR[3], resolving the ocean on a GR15L40 grid, i.e. with a nominal horizontal resolution of 1.5° on 40 depth levels. An oceanic localized Ensemble Kalman Filter, implemented with the Parallel Data Assimilation Framework[4], assimilates monthly EN4 temperature and salinity profiles. Simultaneously, atmospheric nudging of ERA40/ERAInterim/ERA5 reanalyses[5,6,7] takes place. All monthly datasets range from January 1958 until October 2020 and present temperature profiles up to a depth of 700 m in 20 depth steps and are publicly available[8] under `http://hdl.handle.net/21.14106/098c6104e3d89943248aa61ff69db972adb3baf6`.
The observational data used is adapted from the EN4 project[9]. It describes a collection of global ocean temperature and salinity profile datasets spanning from 1900 until present day. It contains observational data from Argo floats, Arctic Synoptic Basinide Oceanography (ASBO), the Global Temperature and Salinity Profile Program (GTSPP) and the World Ocean Database (WOD13)[9]. As these datasets partly overlap, input data is preprocessed and duplicates sorted out before the complete EN4 dataset is created. The same procedure is applied to profiles that are not necessarily identified to be the same but that are very close to each other in time and space[9]. Additionally, various quality control checks are performed in order to identify data profiles with faulty location or measurement data, such as missing depth steps or the location assignment to a point on land rather than in the ocean. The EN4 temperature profiles are remapped to fit the MPI-ESM-grid by binning them to a 0.2°x0.2°xL40 grid before bilinear remapping to the Ocean-GR15L40 grid[10]. Thus, they possess the same vertical and horizontal resolution as the assimilation reanalysis. All EN4 profiles are brought together in NetCDF files and are publicly available under `https://www.metoffice.gov.uk/hadobs/`[9].
For later evaluation we also use the EN4 objective analysis as an independent baseline to the assimilation reanalysis and the NN reconstructions. For the EN4 objective analysis[9], a persistence-based forecast of the oceanic state from the previous month is combined with actual oceanic temperature and salinity profiles from the current month using optimal interpolation. For details of the EN4 analysis scheme, we refer to Good et al. (2013).

Data Preprocessing. Using climate data operators (CDO), all data assimilation reanalysis members are remapped to a 128 x 128 irregular model grid of the North Atlantic (-65 – -5°W, 5 – 69°N). In order to provide an accurate reconstruction, we would like our NN not to learn simply the monthly climatology but to be trained on and reconstruct monthly anomalies. Thus, the assimilation reanalysis is converted to monthly anomaly fields by subtracting its own monthly climatology for each individual month of the year and each gridcell during the Argo era. Due to the lower uncertainty during that time, we can also assume the climatology to be a more accurate description of its seasonal cycle. In order to keep a consistent input for the NN reconstruction, the EN4 observations are converted to anomalies using the same Argo climatology of the data assimilation's 3D temperature reanalysis. Afterwards, the data is read out from NetCDF4 files and converted into HDF5 Files in the correct input shape for each one of the neural network's depth layers. The training input then consists of the assimilation reanalysis and a binary mask marking the places of observations. Additionally, a corresponding masked assimilation reanalysis, meaning the assimilation reanalysis multiplied with the binary observations mask, is produced as an input of the NN training. For the final reconstruction of the trained neural network, the masked assimilation reanalysis in the NN input is replaced by the real EN4 observations.
Of the 16 data assimilation members, two are excluded from training entirely and held back for later independent testing. Thus, the training data is made up of 14 data assimilation members of 202 images each (12 months for the training time period January 2004 – October 2020),

resulting in 2.262 training samples. Every 5th month (566 samples) is used for validation during the training process.

Neural Network Architecture. The neural network adopted in this work is a partial convolutional U-net based on the one used by[11] to reconstruct near-surface air temperature data. Originally, this convolutional neural network was developed for the inpainting of irregularly shaped holes and has outperformed a number of other machine learning based inpainting techniques[12]. The loss function of the network is designed to adress several error sources. On the one, two mean error terms govern the development of the network at the point with and without input. Additionally, a total variation and style terms try to ensure smooth transitions and overall pattern correlation. The detailed code of the loss function can be further explored under: `https://github.com/slenta/Physics-Informed-CNN-Reconstructions`. The network architecture from[11] has been supplemented by the following changes and is graphically depicted in Fig. S1:

- The two dimensional structure for reconstructing SSTs has been enhanced by a third dimension for in-depth subsurface temperatures
- The hyperparameters have been tuned specifically for this application of NA SPG OHC reconstruction
- Postprocessing steps for the calculation of OHC and uncertainty calculation
- Preprocessing for the variable choice of region utilized for training and evaluation
- Possible implementation of a long-short-term memory (LSTM) module
- Variability in shape of the input data (from set 72x72) to any input size
- Various plotting and evaluation possibilities added.

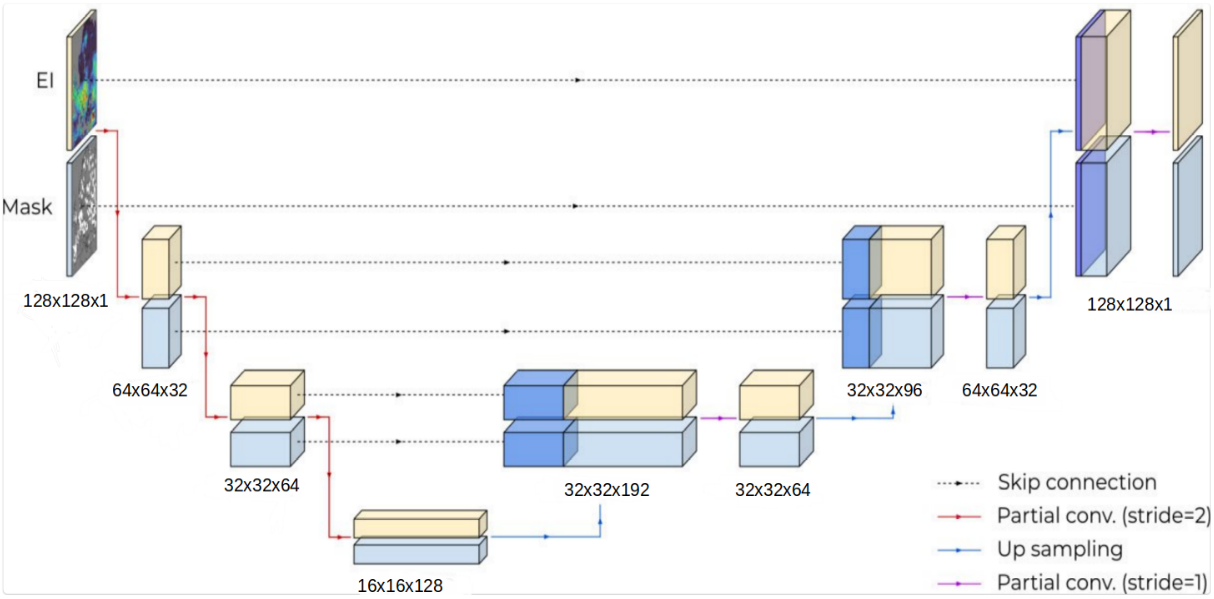

Fig.S1 | Basic architecture underlying the partial convolutional neural network developed by Liu et al. (2018)[12].. The partial convolutional layers are supplemented with max pooling layers and cross connections between encoding and decoding branches. For the application in this manuscript, the network has been adjusted by hyperparameter training, variability of input sizes as well as various pre and postprocessing steps.

The network is additionally supplemented with an observational bias physics-informed approach[13]. This means that a careful selection of training data is conducted in order to teach the neural network realistic physical patterns. Through the temporal division of the assimilation reanalysis and the selection of the more accurate Argo era reanalysis as training data, we can teach the neural network the training data's underlying realistic physics. Some of the network's features, such as the LSTM module have however not been in implemented in the result production for this paper. For that it remains in the shape of the partial convolutional U-net displayed in Fig. S1. In comparison to regular convolutional U-nets, partial convolutions rely on a masked and re-normalized convolution operation, which is then followed by an automatic mask-update step. Through this stepwise infilling of individual gridcells, partial convolutions show excellent results in infilling irregularly shaped holes[12].

Data Postprocessing. The trained neural network is given EN4 3D temperature profiles of the entire period from 1958–2004 for reconstruction. After the infilling, the resulting NN reconstruction is cut to just the NA SPG (-60 – -10°W, 45 – 60°N) for the remaining evaluation. Due to the entire NA being reconstructed no further postprocessing to correct for edge artifacts is necessary. In order to exclude the effects of the base climatology from the Argo era (January 2004 – October 2020), the reconstructed anomalies are kept as such without converting them back to full values. Only for specific plotting purposes (for example Fig. 4), where the NAC flow is relevant, temperature values are converted back to full values by adding the same Argo climatology again. Afterwards, the individual temperature fields of the NN reconstructions are combined to a 2-dimensional OHC field using the following formula:

$$H = \rho_{sw} \cdot \theta_{sw} \sum_{0}^{z_0} T_z \cdot \Delta_z. \quad (1)$$

Here ρ_{sw} denotes the density of seawater, $theta_{sw}$ the specific heat capacity of seawater and T_z the temperature at depth z and Δ_z the thickness of the corresponding vertical level. T_z are kept as anomaly fields for the analysis, in order to better review structural differences in the OHC anomaly fields. The sum is taken from the sea surface up to a depth z_0 = 700 m.

For evaluation purposes, the OHC fields undergo r.s.m.e calculation and timeseries correlation. Additionally, a spatial correlation of the SPG OHC patterns was conducted in order to test for both temporal and spatial correlation between NN reconstructions and assimilation reanalysis.

Code. All relevant code with explanations can be found and copied under `https://github.com/slenta/Physics-Informed-CNN-Reconstructions`. However, the code is subject to ongoing changes and will be updated continually. To ensure reproducibility of our results, the version utilized in this manuscript has been saved in the branch Lentzetal2024PINN.

Appendix B Assimilation Reanalysis Bias during the Argo era

An important feature of the Argo era NN reconstructions is a systematic negative bias during the assimilation reanalysis' OHC peak during the beginning of the 2000s. We assume that the NN reconstruction's disagreement with the assimilation reanalysis is not a sign of a lack of quality. Instead, we think that several factors support the NN's estimate of a lower peak. On the one hand this assumption is supported by the general data assimilation bias at the points of observations. During the Argo era, but in particular during this peak, the assimilation possesses a systematic positive bias compared to the observations. The NN reconstructions follow the observations much closer than the assimilation reanalysis. This systematic bias is shown in the following Fig. S2:

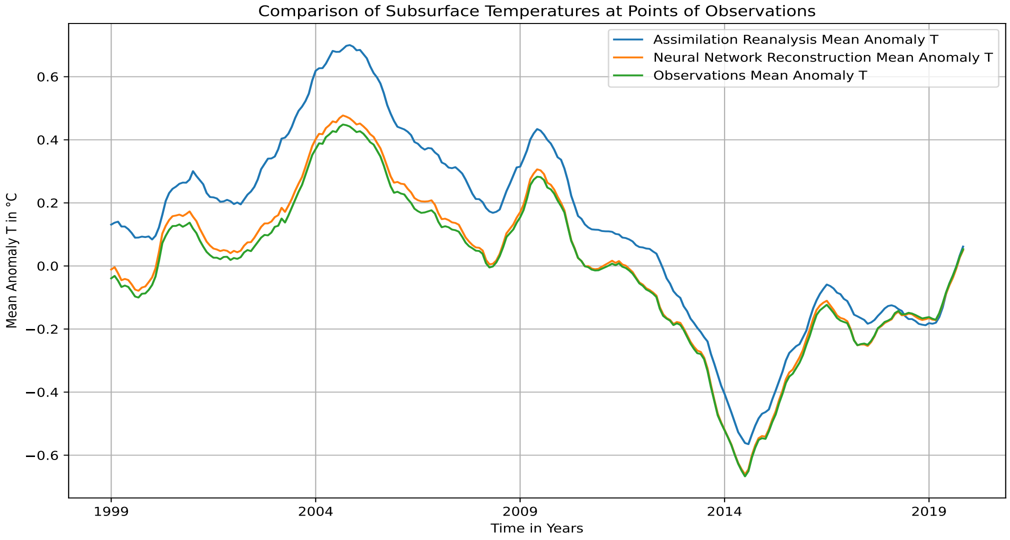

Fig.S2 | Comparison of NN reconstruction, observations and assimilation reanalysis at the points of observations. The shown data consists of mean anomaly subsurface temperatures at all gridpoints with EN4 observations supplemented by an annual running mean.

Additional support for this statement can be gained by comparing the NN reconstruction and assimilation reanalysis OHC timeseries with other OHC estimates (Fig. S3). On the one hand, a comparison of our direct NN reconstructions of the observational fields (yellow line and the output data utilized in the remaining analysis until now) with a NN reconstruction, which uses the masked assimilation reanalysis points as inputs instead of the observational values, (green line) is shown. This proves that with the same values at the input points as the assimilation reanalysis, the NN is able to reproduce the OHC estimate of the assimilation reanalysis (blue line) more closely. On the other hand, the shown EN4 objective analysis[9] (red line) also supports a lower peak during this start of the Argo era:

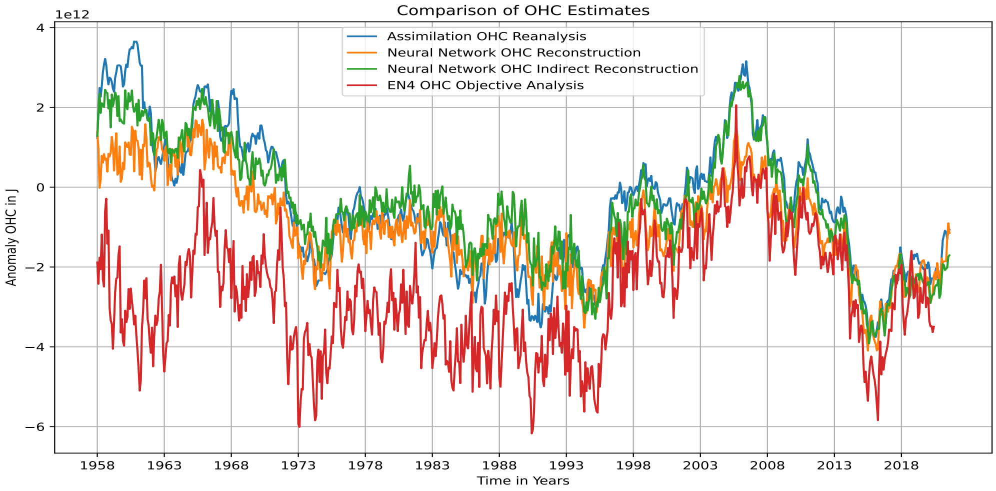

Fig.S3 | Comparison of NN reconstruction of observations (orange) and masked assimilation (green), assimilation reanalysis (blue) and EN4 objective analysis (red) SPG anomaly OHC. All estimates represent the monthly OHC values fo the NA SPG and have been converted to anomalies by subtracting the same assimilation reanalysis monthly Argo climatology.

Appendix C Detailed recording of monthly NN OHC reconstructions

In the following, exemplary images to compare NN OHC reconstructions and the assimilation OHC reanalysis are given (Fig. S4-S9). In order to put focus on the regional structures of the estimates, we provide anomaly OHC estimates by both methods. For demonstrative purposes, the examples are chosen to cover a range of different months, years and states of the NAC northwest corner in both pre-Argo and Argo era. During the pre-Argo era differences in the northwest corner regions are clearly visible, with the NN reconstructions not showing the typical negative bias of the assimilation reanalysis. This bias, artifact of the incorrect model NAC flow, is however not always visible, only if there are no observations in the relevant region. During the Argo era, there are much greater similarities between both OHC estimates with the assimilation reanalysis not showing the typical negative bias.

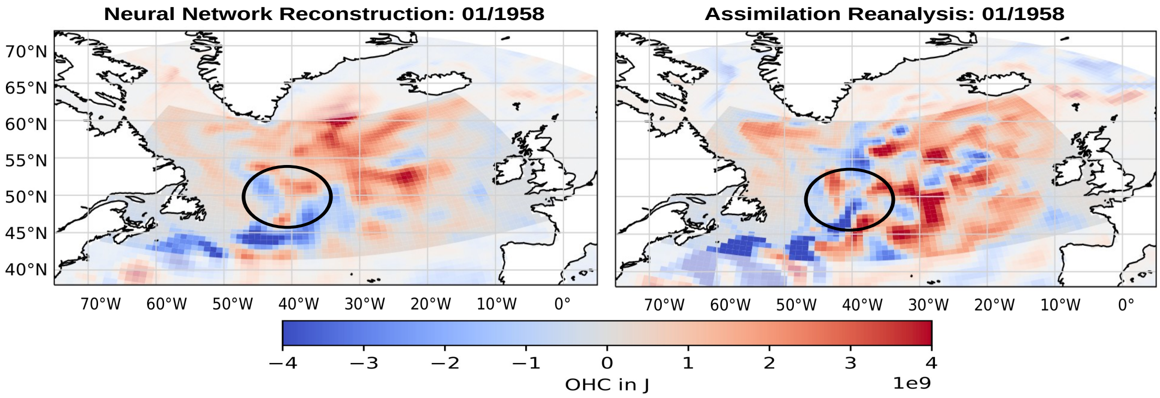

Fig.S4 | Example comparison of monthly anomaly OHC estimate by NN reconstruction and assimilation analysis. Anomaly OHC estimates for January 1958. The region of the NAC's northwest corner is marked by the black circle.

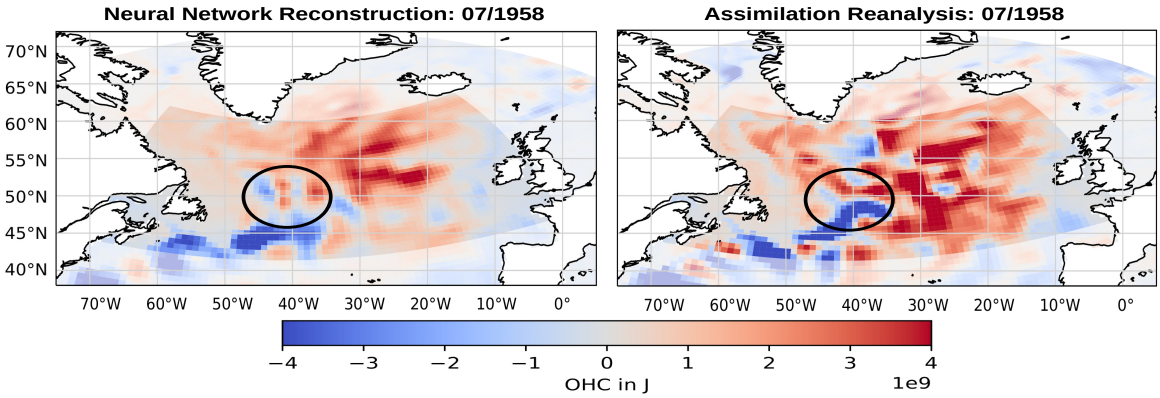

Fig.S5 | Example comparison of monthly anomaly OHC estimate by NN reconstruction and assimilation analysis. Anomaly OHC estimates for July 1958. The region of the NAC's northwest corner is marked by the black circle.

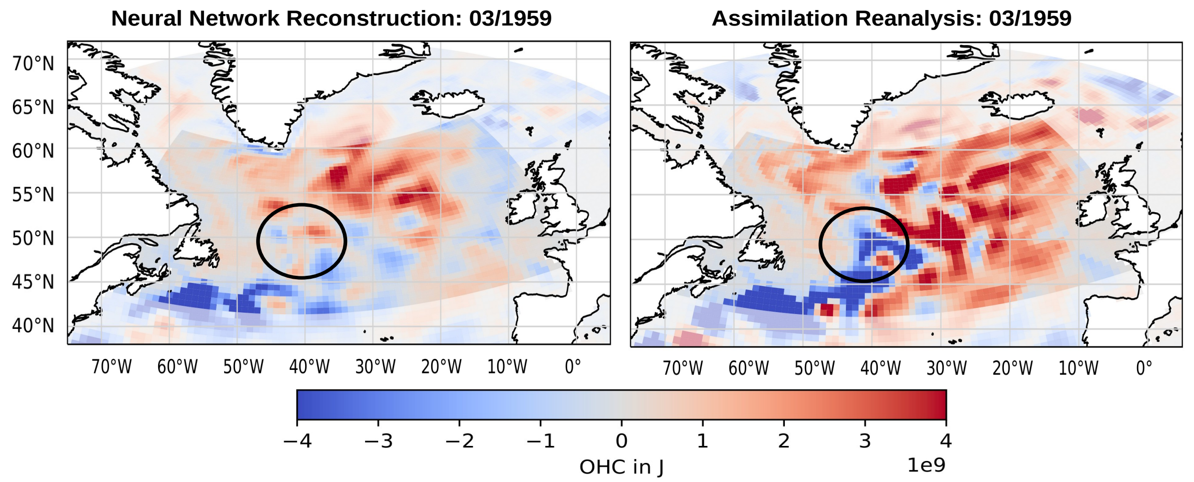

Fig.S6 | Example comparison of monthly anomaly OHC estimate by NN reconstruction and assimilation analysis. Anomaly OHC estimates for March 1959. The region of the NAC's northwest corner is marked by the black circle.

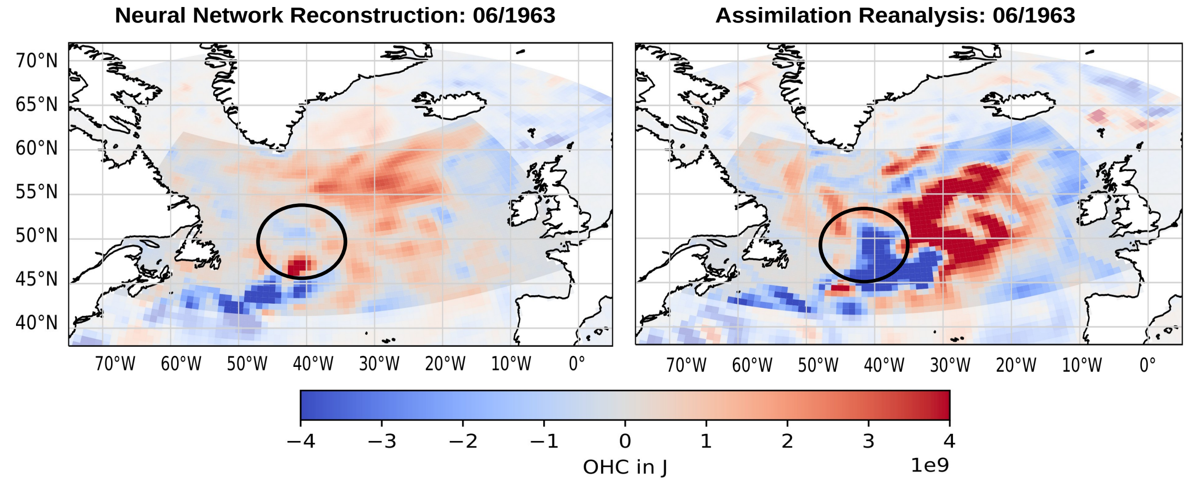

Fig.S7 | Example comparison of monthly anomaly OHC estimate by NN reconstruction and assimilation analysis. Anomaly OHC estimates for June 1963. The region of the NAC's northwest corner is marked by the black circle.

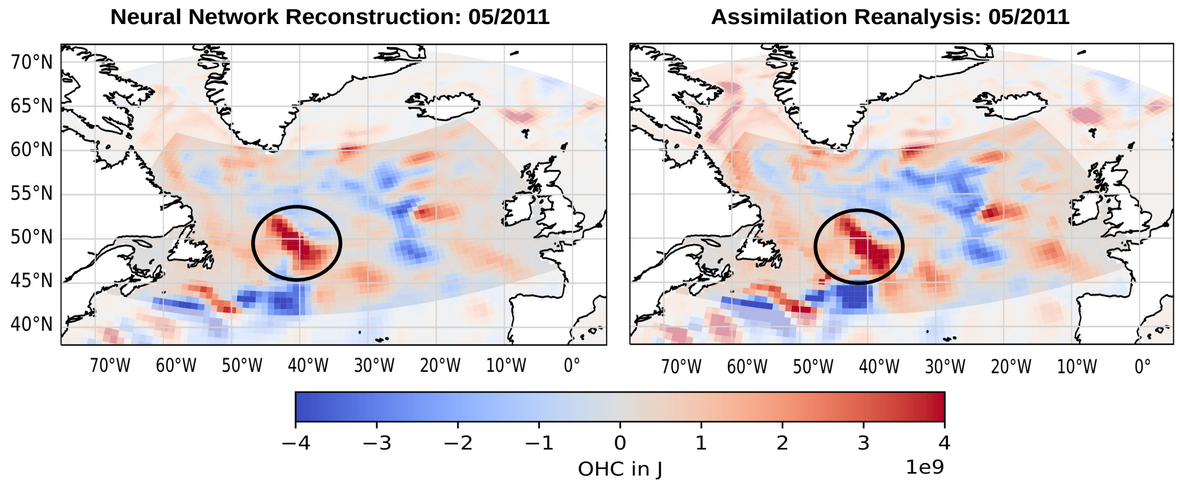

Fig.S8 | Example comparison of monthly anomaly OHC estimate by NN reconstruction and assimilation analysis. Anomaly OHC estimates for May 2011. The region of the NAC's northwest corner is marked by the black circle.

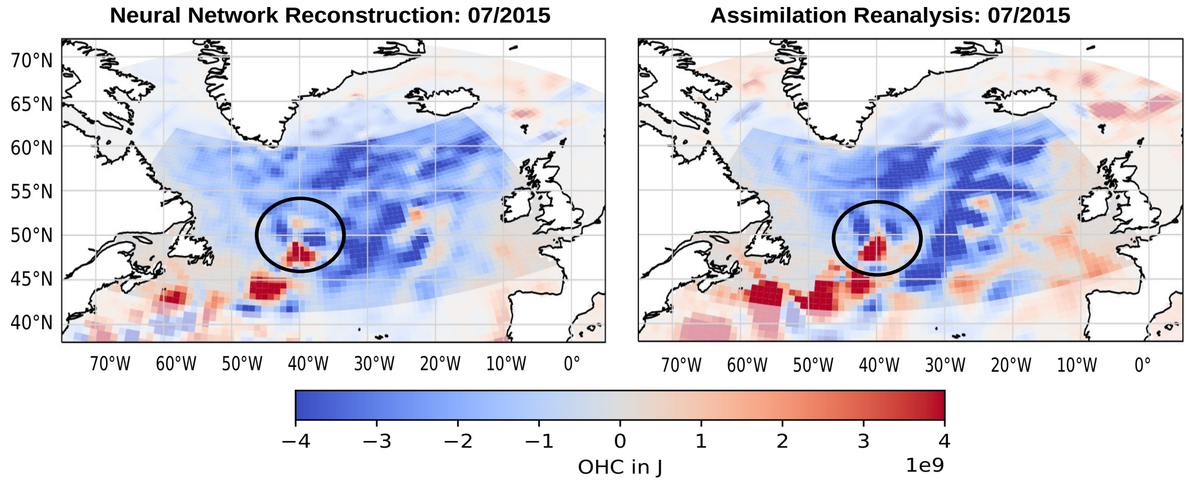

Fig.S9 | Example comparison of monthly anomaly OHC estimate by NN reconstruction and assimilation analysis. Anomaly OHC estimates for January 2018. The region of the NAC's northwest corner is marked by the black circle.

Appendix D Assimilation Reanalysis NAC Northwest Corner Bias

The utilized method by this NN assumes that the learned patterns from the Argo era are applicable in pre-Argo era reconstructions. Fig. S10 shows that SST observations in the northwest corner region (48 - 53 °N, 40 - 45 °W) do not show significantly different ranges or patterns in the pre-Argo era. Instead, it is very clear that the assimilation reanalysis jumps to a negative bias, whenever there are not enough observations available, e.g. 1960 – 2000 and in particular the 1970s. Concluding from this analysis, it becomes clear that the NN reconstructions do not wrongly assume stationary boundary conditions. Instead, the assimilation reanalysis reverts to a biased state of its base model when observations do not indicate otherwise.

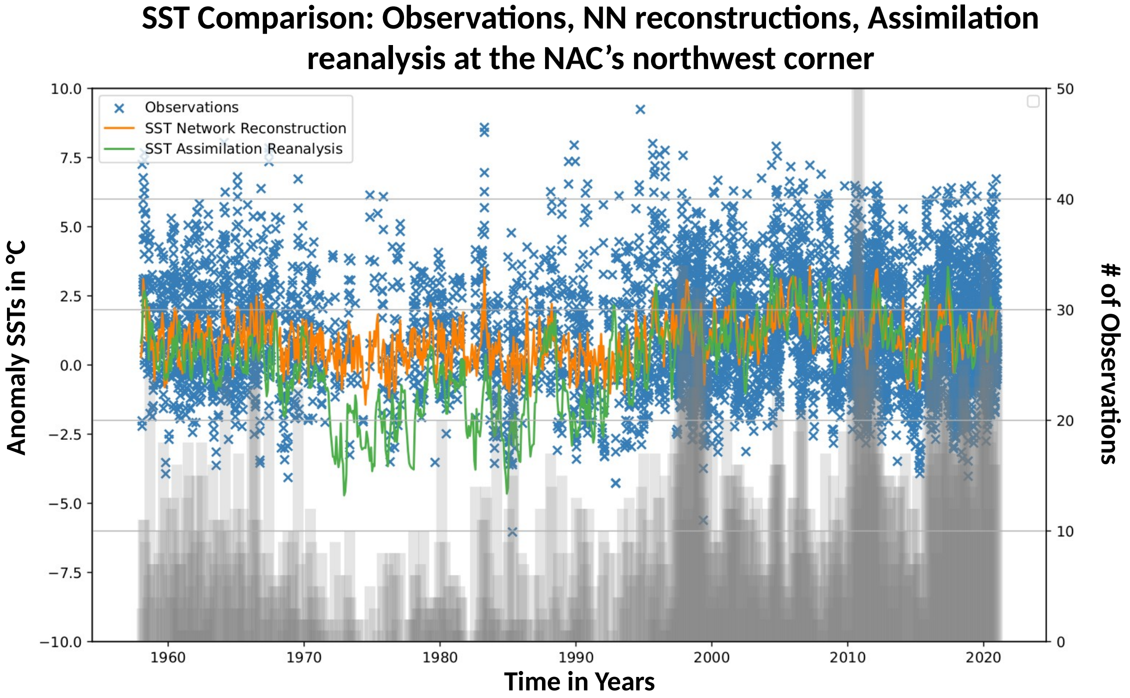

Fig.S10 | SST timeseries comparison of the NN reconstruction (orange) and assimilation reanalysis (green) in the NAC's northwest corner region. Additionally, all available EN4 SST observations in the area of the NAC's northwest corner are marked by blue crosses. The total amount of observations in the region is shown by the grey bars.

Appendix E Result Evaluation: Comparison to EN4 Objective Analysis

In the case of subsurface temperatures or ocean heat content, evaluation of reconstructions during the pre-Argo era cannot not be conducted with observational data due to the lack thereof. Thus, we can only compare results with independent other estimates. For that comparison we choose the EN4 objective analysis[9] (extensive description in section A).

We compare the EN4 objective analysis with the assimilation reanalysis and our NN reconstructions through the calculation of the root mean squared error (RMSE - Fig. S11) and the PearsonR correlation coefficient (Fig. S12) for each grid box.

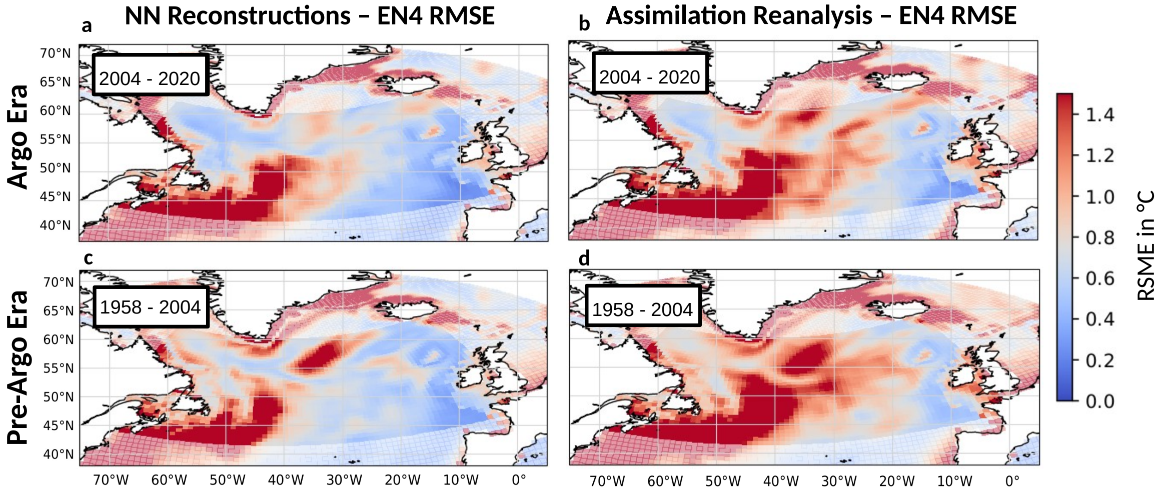

Fig.S11 | RMSE of EN4 objective subsurface temperature analysis with the NN 3d temperature reconstructions and the assimilation 3d temperature reanalysis. The error is calculated for subsurfaces temperatures and given in °C in order to give an intuitive measure for the differences. The high errors in the wester NAC region are normal due to the unpredictable nature of the far western NAC.

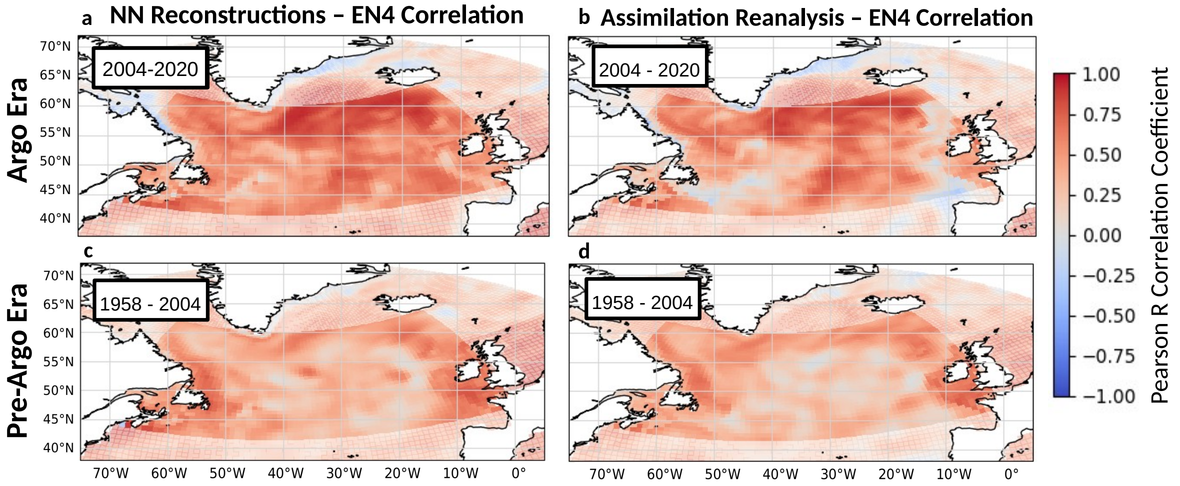

Fig.S12 | Gridcellwise PearsonR correlation coefficient of EN4 objective OHC analysis with the NN OHC reconstructions and the assimilation OHC reanalysis.

The evaluation with an independent baseline shows two important aspects. On the one hand, the NN reconstructions show generally similar patterns and behavior as its state-of-the-art training assimilation reanalysis. Also outside of its training period (the Argo era), the NN reconstructions do not show significantly higher biases or errors. On the other hand, the NN reconstructions show generally slightly higher correlations and lower RMSE with the EN4 objective analysis compare to the assimilation data analysis. This lower error is especially pronounced in the areas, where we know the assimilation reanalysis to possess significant biases, such as the NAC's northwest corner region. Therefore, this evaluation supports our conclusion that the NN reconstructions show generally similarly realistic physical patterns as its training data, but

improves on certain errors of the assimilation reanalysis that are especially pronounced in the pre-Argo era.

Appendix F Detailed OHC estimate for NN reconstructions and assimilation reanalysis in Argo and pre-Argo era

As Fig.4 of the main manuscript only shows a very dual plot tuned to highlight the NAC's northwest corner, Fig. S13 provides the same data but with a colorbar that allows for a more detailed depiction.

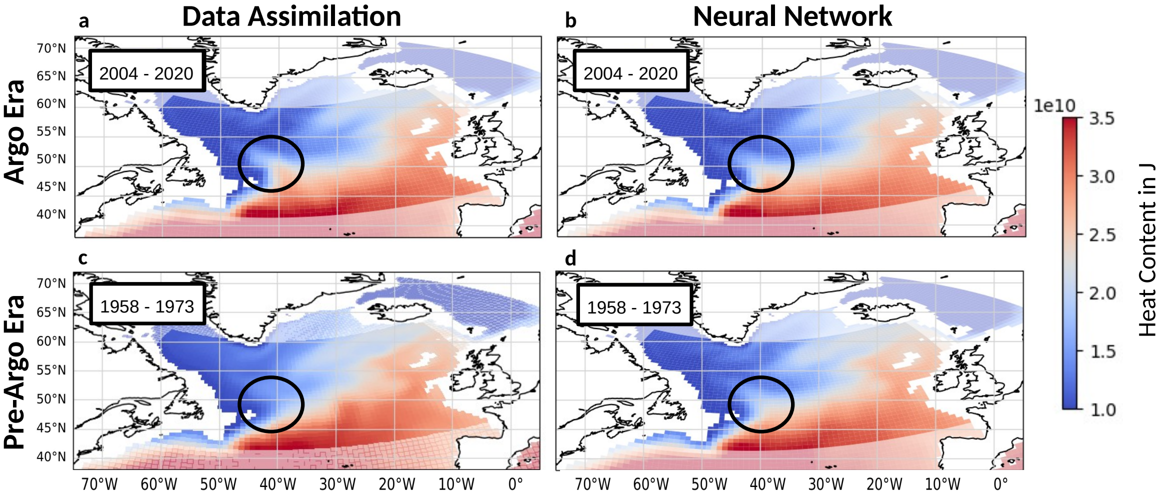

Fig.S13 | Assimilation reanalysis and NN reconstruction of the North Atlantic Current's northwest corner. All OHC estimates are calibrated to show the NAC flow as the gradient between the red and blue areas, while the black circles show the region of the NAC northwest corner. Data assimilation (a) and NN reconstructions (b) during the later Argo era (2004 – 2020) show a sharp corner in the northwest of the NAC. In a comparable mean during the pre-Argo era (1958 – 1973), the data assimilation (c) misses this corner, while the NN still shows the characteristic sharp right turn (d).

Appendix G List of Acronyms

OHC Ocean Heat Content

PINN Physics-informed Neural Network

TL Transfer Learning

ACC Anomaly Correlation Coefficient

MPI Max Planck Institute

NA North Atlantic

NN Neural Network

SPG Subpolar Gyre

NAC North Atlantic Current

SST Sea Surface Temperature

ESM Earth System Model

List of Figures

S1. NN neural network architecture 2
S2. Comparison of NN reconstruction, observations and assimilation reanalysis at the points of observations 4
S3. Comparison of NN reconstruction of observations and masked assimilation, assimilation reanalysis and EN4 objective analysis SPG anomaly OHC 5
S4. NN reconstruction example 1 6
S5. NN reconstruction example 1 6
S6. NN reconstruction example 1 7
S7. NN reconstruction example 1 7
S8. NN reconstruction example 1 8
S9. NN reconstruction example 1 8
S10. SST Comparison Northwest Corner 9
S11. RMSE EN4 Objective Analysis with NN Reconstructions and Assimilation Reanalysis 10
S12. PearsonR Correlation EN4 Objective Analysis with NN Reconstructions and Assimilation Reanalysis 10
S13. Assimilation reanalysis and NN reconstruction of the North Atlantic Current's northwest corner during Argo and pre-Argo era 11

1. References

[1] S. Brune and J. Baehr. “Preserving the coupled atmosphere–ocean feedback in initializations of decadal climate predictions”. en. In: *WIREs Climate Change* 11.3 (May 2020) (cit. on p. 1).

[2] L. Hövel, S. Brune, and J. Baehr. “Decadal Prediction of Marine Heatwaves in MPI-ESM”. en. In: *Geophysical Research Letters* 49.15 (Aug. 2022), e2022GL099347 (cit. on p. 1).

[3] T. Mauritsen et al. “Developments in the MPI-M Earth System Model version 1.2 (MPI-ESM1.2) and Its Response to Increasing CO_2”. In: *Journal of Advances in Modeling Earth Systems* 11.4 (Apr. 2019), pp. 998–1038 (cit. on p. 1).

[4] L. Nerger and W. Hiller. “Software for ensemble-based data assimilation systems—Implementation strategies and scalability”. en. In: *Computers & Geosciences* 55 (June 2013), pp. 110–118 (cit. on p. 1).

[5] S. M. Uppala et al. “The ERA-40 re-analysis”. en. In: *Quarterly Journal of the Royal Meteorological Society* 131.612 (Oct. 2005), pp. 2961–3012 (cit. on p. 1).

[6] D. P. Dee et al. “The ERA-Interim reanalysis: configuration and performance of the data assimilation system”. en. In: *Quarterly Journal of the Royal Meteorological Society* 137.656 (Apr. 2011), pp. 553–597 (cit. on p. 1).

[7] H. Hersbach et al. “The ERA5 global reanalysis”. en. In: *Quarterly Journal of the Royal Meteorological Society* 146.730 (July 2020), pp. 1999–2049 (cit. on p. 1).

[8] S. Brune et al. *MPI-ESM-LR_1.2.01p5 decadal predictions localEnKF: monthly mean values.* 2021 (cit. on p. 1).

[9] S. A. Good, M. J. Martin, and N. A. Rayner. “EN4: Quality controlled ocean temperature and salinity profiles and monthly objective analyses with uncertainty estimates: THE EN4 DATA SET”. en. In: *Journal of Geophysical Research: Oceans* 118.12 (Dec. 2013), pp. 6704–6716 (cit. on pp. 1, 4, 9).

[10] S. Brune, L. Nerger, and J. Baehr. “Assimilation of oceanic observations in a global coupled Earth system model with the SEIK filter”. en. In: *Ocean Modelling* 96 (Dec. 2015), pp. 254–264 (cit. on p. 1).

[11] C. Kadow, D. M. Hall, and U. Ulbrich. “Artificial intelligence reconstructs missing climate information”. en. In: *Nature Geoscience* 13.6 (June 2020), pp. 408–413 (cit. on p. 2).

[12] G. Liu et al. “Image Inpainting for Irregular Holes Using Partial Convolutions”. en. In: *arXiv:1804.07723 [cs]* (Dec. 2018). arXiv: 1804.07723 (cit. on pp. 2, 3).

[13] G. E. Karniadakis et al. “Physics-informed machine learning”. en. In: *Nature Reviews Physics* 3.6 (May 2021), pp. 422–440 (cit. on p. 3).
